# Biomarkers in long COVID-19: A systematic review

**DOI:** 10.3389/fmed.2023.1085988

**Published:** 2023-01-20

**Authors:** Yun-Ju Lai, Shou-Hou Liu, Sumatchara Manachevakul, Te-An Lee, Chun-Tse Kuo, Dhimiter Bello

**Affiliations:** ^1^School of Nursing, Zuckerberg College of Health Sciences, University of Massachusetts Lowell, Lowell, MA, United States; ^2^Institute of Biomedical Sciences, Academia Sinica, Taipei, Taiwan; ^3^Graduate Institute of Microbiology, College of Medicine, National Taiwan University, Taipei, Taiwan; ^4^Department of Biomedical and Nutritional Sciences, Zuckerberg College of Health Sciences, University of Massachusetts Lowell, Lowell, MA, United States

**Keywords:** biomarker, long COVID, IL-6, CRP, TNF-α

## Abstract

**Purpose:**

Long COVID, also known as post-acute sequelae of COVID-19, refers to the constellation of long-term symptoms experienced by people suffering persistent symptoms for one or more months after SARS-CoV-2 infection. Blood biomarkers can be altered in long COVID patients; however, biomarkers associated with long COVID symptoms and their roles in disease progression remain undetermined. This study aims to systematically evaluate blood biomarkers that may act as indicators or therapeutic targets for long COVID.

**Methods:**

A systematic literature review in PubMed, Embase, and CINAHL was performed on 18 August 2022. The search keywords long COVID-19 symptoms and biomarkers were used to filter out the eligible studies, which were then carefully evaluated.

**Results:**

Identified from 28 studies and representing six biological classifications, 113 biomarkers were significantly associated with long COVID: (1) Cytokine/Chemokine (38, 33.6%); (2) Biochemical markers (24, 21.2%); (3) Vascular markers (20, 17.7%); (4) Neurological markers (6, 5.3%); (5) Acute phase protein (5, 4.4%); and (6) Others (20, 17.7%). Compared with healthy control or recovered patients without long COVID symptoms, 79 biomarkers were increased, 29 were decreased, and 5 required further determination in the long COVID patients. Of these, up-regulated Interleukin 6, C-reactive protein, and tumor necrosis factor alpha might serve as the potential diagnostic biomarkers for long COVID. Moreover, long COVID patients with neurological symptoms exhibited higher levels of neurofilament light chain and glial fibrillary acidic protein whereas those with pulmonary symptoms exhibited a higher level of transforming growth factor beta.

**Conclusion:**

Long COVID patients present elevated inflammatory biomarkers after initial infection. Our study found significant associations between specific biomarkers and long COVID symptoms. Further investigations are warranted to identify a core set of blood biomarkers that can be used to diagnose and manage long COVID patients in clinical practice.

## Introduction

The coronavirus disease (COVID-19) was defined as an infectious disease caused by the SARS-CoV-2 virus (1). While the majority of people recovered fully from COVID-19, 45% of COVID survivors might suffer from a variety of unresolved symptoms, which persisted for nearly 4 months after SARS-CoV-2 infection and are referred to as long COVID (2). Older adults might be less likely to experience long COVID than younger adults (3). Additionally, the incidence of experiencing long COVID symptoms post-infection is significantly greater among women versus men (4).

Long COVID manifests as a complex set of symptoms, including neurological, neuropsychiatric, cardiopulmonary, and gastrointestinal (3). Across the studies that have reported the prevalence of long COVID symptoms, among the neurological and neuropsychiatric symptoms more frequently associated with long COVID are fatigue (29–58%), headache (10–44%), and anxiety or depression (22–28%) (5–8). Shortness of breath or difficulty breathing (21–24%) and loss of taste or smell (12–15%) are also frequently reported by long COVID patients with pulmonary symptoms (7, 8). Interestingly, in patients who experienced long COVID syndrome, neurological, neuropsychiatric, cardiopulmonary, and gastrointestinal, and other complications (primarily rheumatological complications) were significantly more likely observed in female than in male patients (9). Furthermore, persistent pulmonary or neurological manifestations seen in long COVID may affect an individual’s ability to perform their work, as well as routine daily living activities, such as household chores (6).

One urgent public health question is how to monitor and relieve these long COVID symptoms (3). In this regard, it is desirable to have access to non- or minimally invasive biomarkers, such as those that are often measured in readily available patient blood samples. Clinically-relevant circulating biomarkers may serve as valuable indicators of patients’ normal physiological conditions or disease severity. For example, the up-regulated levels of neurofilament light chain (NFL) and glial fibrillary acidic protein (GFAP) in serum may indicate neuronal damage in the progression of neurodegenerative diseases, such as Alzheimer’s disease (10) or Parkinson’s disease (11). In addition, Interleukin (IL) 6 was not only identified as a prognostic biomarker for disease monitoring in cancer patients with severe COVID-19 (12) but also served as a target for treating COVID-19-related systemic inflammation, such as acute respiratory distress syndrome and cytokine release syndrome (13, 14). While the literature on this topic is evolving fast, to-date diagnostic biomarkers for long COVID remain unclear. This study aims to systematically evaluate the published peer-reviewed literature with the goal of identifying blood biomarkers that may serve as indicators or therapeutic targets for long COVID.

## Materials and methods

### Search strategy

A systematic literature review in PubMed, Embase, and CINAHL was performed on 18 August 2022. The publication time limit for this search was not specified in order to capture all relevant literature. The search keywords included two broad categories of (1) long COVID-19 symptoms and (2) biomarkers, each with a defined yet broad subset of keywords, as documented in Supplementary Table 1. Duplicate records retrieved from these three databases were removed. Then, all relevant articles from reference lists were identified. Articles must be available in full text. After screening the titles and abstracts, final eligibility is determined based on the full content.

### Eligibility criteria

The inclusion criteria for this systematic review were as follows: (1) Types of study: the primary source of quantitative studies in a peer-reviewed journal published in English. All original studies, including randomized or non-randomized controlled clinical trials, case reports/case series, and correspondences, were included. For mixed-method studies, if quantitative data could be extracted separately, the studies were included. (2) Types of participants: adult long COVID patients were allowed. No restrictions were imposed on the participants’ sex, ethnicity, and clinical symptoms. (3) Types of outcome measures: biomarker data were reported. Articles that did not provide biomarkers or did not have statistically significant data were excluded. Unpublished theses, dissertations, review articles, conference proceedings, and studies using animal models were also excluded.

### Data extraction

According to the inclusion and exclusion criteria, data extraction was completed by two authors (SM and S-HL) and verified by another author (Y-JL). The following headlines were extracted from the articles: authors, study location, number of total patients, patients age (median/mean), long COVID timeframe, comparison groups, types of symptoms, biomarker measurements, and conclusions (Supplementary Table 2).

### Quality assessment

The quality of the studies was assessed using the modified REporting recommendations for tumor MARKers prognostic studies (REMARK), which provides a valuable reference when reporting or analyzing medical studies related to diseases markers or prognostic markers (12, 15, 16). Two independent reviewers (S-HL and T-AL) verified the total scores. The percentages of studies reviewed that met the criteria for methodological quality are shown in Supplementary Table 3, and outcomes were summarized in the respective section of results.

## Results

### Characteristics of the studies

**FIGURE 1:**
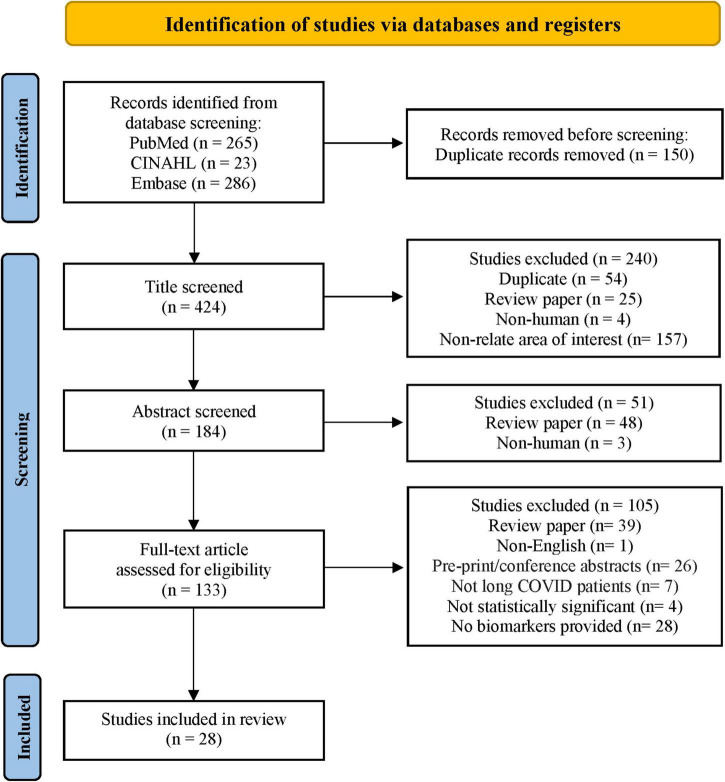
Flow diagram of the literature search. Adapted from Page et al. (17).

Through the database search, 574 studies from PubMed, CINAHL, and Embase were identified. The search process adhered to the Preferred Reporting Items for Systematic Reviews and Meta-Analyses (PRISMA) (17) flow diagram, as shown in Figure 1. After screening each article’s title and abstract, 133 full-text articles were assessed for eligibility. Twenty-eight studies met the eligibility criteria and were included in this systematic review. The 28 articles listed in ascending alphabetical order (Supplementary Table 2) were published between 2021 and 2022. The eligible studies were conducted in the United States (9, 32.1%), Spain (4, 14.3%), Italy (3, 10.7%), Germany (2, 7.1%), and 1 (3.6%) each in Australia, Brazil, Colombia, Costa Rica, Egypt, India, Ireland, Mexico, Singapore, and Turkey. Among 3,374 participants, 1,569 (46.5%) were long COVID patients, 1,419 (42.1%) were participants who completely recovered from COVID-19, 255 (7.6%) were healthy participants (vaccinated and unvaccinated), and 104 (3.1%) were patients with COVID-19. Of 193 biomarkers tested in the 28 studies, 113 (58.5%) were significantly associated with long COVID symptoms. Long COVID timeframe was defined according to definitions used in the reviewed articles: less than 3 months after SARS-CoV-2 infection in 8 (28.6%) studies, 3–6 months in 9 (32.1%) studies, and 6 or more months in 3 (10.7%) studies. There is one (3.6%) study with a various range (22 to 322 days), and the rest 7 (25%) studies did not provide the definition.

### Methodological assessment

**FIGURE 2:**
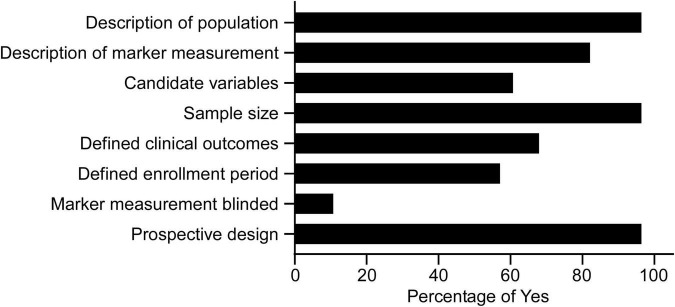
Study quality of the 28 articles in the systematic review assessed by the modified REMARK questionnaire (Supplementary Table 3).

All the eligible studies were further evaluated by the modified REMARK questionnaire (18). As shown in Supplementary Table 3 and Figure 2, the majority of studies used a prospective design (96.4%), provided a rationale for the sample sizes (96.4%), described the characteristics of the study population (96.4%), and provided information on the measurement of biomarkers (82.1%). 67.9% defined clinical outcomes, 60.7% provided a list of candidate variables, and 57.1% defined patients’ enrollment period. Few articles blinded the measurements of biomarkers to patient outcomes (10.7%).

## Biomarker findings

### Biomarkers related to biological functions

Among 113 biomarkers, 69.9% (79 of 113) biomarkers were significantly increased, 25.7% (29 of 113) biomarkers were decreased, and 4.4% (5 of 113) biomarkers required further determination in long COVID patients. To facilitate the understanding of biological mechanisms in long COVID related biomarkers, the biomarkers were divided into six categories based on their biological function: (1) Cytokines/Chemokines (38, 33.6%); (2) Biochemical markers (24, 21.2%); (3) Vascular markers (20, 17.7%); (4) Neurological markers (6, 5.3%); (5) Acute phase protein (5, 4.4%); and (6) Others (20, 17.7%) (Table 1). With respect to immune response, long COVID patients exhibited higher levels of pro-inflammatory cytokines/chemokines [IL-6, tumor necrosis factor alpha (TNF-α), IL-17, IL-4, and C-C motif chemokine ligand (CCL) 2] and acute phase proteins [C-reactive protein (CRP) and ferritin]. For biochemical markers associated with metabolism, COVID-19 patients with elevated levels of lactate dehydrogenase (LDH) tended to experience long COVID symptoms. Furthermore, in terms of neurological and vascular markers, patients with increased NFL and vascular endothelial growth factor (VEGF) plus decreased hemoglobin showed worse long COVID symptoms.

**TABLE 1:** Categories of biomarkers significantly associated with long COVID symptoms.

Category	Biomarker	References
Acute phase protein	Albumin, C5b-9, CRP, Ferritin, Fibrinogen	(20, 22, 40–45)
Biochemical marker	1-Methylnicotinamide, 2-Phenylphenol, 3,5-Dihydroxybenzoic acid, ADA, ALT, AST, β-glucan, CPA3, Glutamine/Glutamate ratio, Indole-3-lactic acid, L-Cystein, LDH, L-Glutamine, L-Methionine, Ornithine, Pipecolic acid, Quinolinic acid, Quinolinic acid/Tryptophan, Sarcosine, S-Sulfocysteine, ST1A1, Taurine, Tryptase, uPA	(22, 42, 46–51)
Cytokine/chemokine	CCL2, CCL3, CCL4, CCL5, CCL7, CCL19, CCL20, CCL23, CXCL1, CXCL9, CXCL10, CXCL11, Flt3L, G-CSF, GM-CSF, IFN-α, IFN-β, IFN-γ, IL-1α, IL-1β, IL-2, IL-4, IL-6, IL-7, IL-10, IL-10Rβ, IL-12β, IL-13, IL-17, IL-18, IL-33, IP-10, M-CSF, SCF, TGF-α, TGF-β, TNF-α, TNF-β	(19, 21, 22, 40, 43, 47, 48, 52–59)
Neurological marker	GDNF, GFAP, NGF-β, NFL, NT-3, pGFAP/pNFL	(19, 20, 48, 60)
Vascular marker	Ang-2, Col1A2, Col3A1, D-dimer, ESR, ET-1, Factor VIII:C, Hemoglobin, MMP-1, MMP-9, MPO, NO, PDGF-BB, sICAM-1, sTM, sVEGFR, sVCAM-1, VEGF, VWF:Ag, VWF:pp	(21, 42, 44, 46, 48, 52, 56, 58, 59, 61–63)
Others	Ab, ARTN, α-SMA, AXIN, CASP-8, CST-5, Cystatin C, Hs TnT, IGFBP-4, LBP, miRNA21, MRP8/14, NGAL, NT-proBNP/NT-BNP, OPG, OSM, SIRT2, STAMBP, TNFRSF9, Zonulin	(21, 48, 49, 51, 54, 58, 59, 63)

A detailed list of abbreviations in Table 1 can be found in Supplementary Table 2.

### Biomarkers for long COVID patients

**FIGURE 3:**
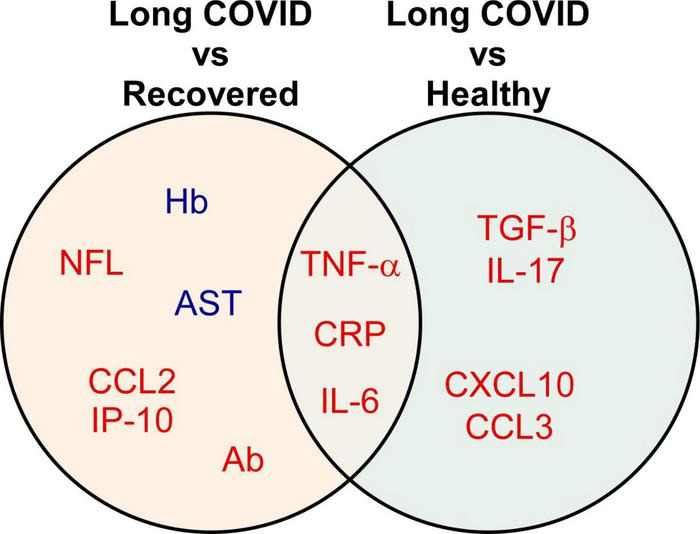
Biomarkers significantly associated with different comparison groups. The Venn diagram presents the 13 biomarkers that were reported by two or more eligible studies. Red indicates up-regulated, while blue refers to down-regulated biomarkers. A detailed list of abbreviations in Figure 3 can be found in Supplementary Table 2.

Among 28 studies, 20 (71.4%) studies reported biomarkers between long COVID and completely recovered patients, and 12 (42.9%) studies demonstrated biomarkers between long COVID patients and healthy participants (Table 2). Compared with recovered COVID patients, long COVID patients showed higher levels of IL-6 (6 of 20, 30%), CRP (3 of 20, 15%), and TNF-α (3 of 20, 15%); lower levels of hemoglobin (2 of 20, 10%). Notably, cytokine/chemokine and biochemical markers accounted for 23.9% (11 of 46) and 39.1% (18 of 46), respectively. Moreover, matched with healthy participants, increased levels of IL-6 (4 of 12, 33.3%), TNF-α (2 of 12, 16.7%), IL-17 (2 of 12, 16.7%), and CCL3 (2 of 12, 16.7%) were associated with long COVID patients. In particular, 44.2% (38 of 86) are cytokine/chemokine, and 20.9% (18 of 86) are vascular markers. The Venn diagram comparison analysis of the differently regulated biomarkers among various groups revealed that IL-6, CRP, and TNF-α remain up-regulated in long COVID patients and may be important indicators of long COVID syndrome (Figure 3).

**TABLE 2:** Biomarkers significantly associated with different comparison groups.

Comparison groups	Categories of biomarkers						References
	Acute phase protein	Biochemical marker	Cytokine/Chemokine	Neurological marker	Vascular marker	Others	
Long COVID vs recovered	Albumin, CRP, Ferritin, Fibrinogen	1-Methylnicotinamide, 2-Phenylphenol, 3,5-Dihydroxybenzoic acid, ALT, AST, β-glucan, Indole-3-lactic acid, LDH, L-Cystein, L-Glutamine, L-Methionine, Ornithine, Pipecolic acid, Quinolinic acid, Quinolinic acid/Tryptophan, Sarcosine, S-Sulfocysteine, Tryptase	CCL2, CXCL10, GM-CSF, IFN-γ, IL-2, IL-4, IL-6, IL-10, IL-17, IP-10, TNF-α	GFAP, NFL, pGFAP/pNFL	ET-1, Hemoglobin, NO, PDGF-BB, VEGF	Ab, Hs TnT, LBP, NT-proBNP/NT-BNP, Zonulin	(19, 20, 40, 41, 43–49, 51, 53, 54, 56–58, 60, 62, 63)
Long COVID vs healthy	Albumin, C5b-9, CRP, Ferritin	ADA, CPA3, Glutamine/Glutamate ratio, LDH, ST1A1, Taurine, Tryptase, uPA	CCL2, CCL3, CCL4, CCL5, CCL7, CCL19, CCL20, CCL23, CXCL1, CXCL9, CXCL10, CXCL11, Flt3L, G-CSF, GM-CSF, IFN-α, IFN-β, IFN-γ, IL-1α, IL-1β, IL-2, IL-4, IL-6, IL-7, IL-10, IL-10Rβ, IL-12β, IL-13, IL-17, IL-18, IL-33, IP-10, M-CSF, SCF, TGF-α, TGF-β, TNF-α, TNF-β	GDNF, NGF-β, NT-3	Ang-2, Col1A2, Col3A1, D-dimer, ESR, ET-1, Factor VIII:C, Hemoglobin, MMP-1, MMP-9, MPO, sICAM-1, sTM, sVCAM-1, sVEGFR, VEGF, VWF:Ag, VWF:pp ARTN, α-SMA, AXIN, CASP-8, CST-5, Cystatin-C, IGFBP-4, miRNA21, MRP8/14, NGAL, OPG, OSM, SIRT2, STAMBP	ARTN, α-SMA, AXIN, CASP-8, CST-5, Cystatin-C, IGFBP-4, miRNA21, MRP8/14, NGAL, OPG, OSM, SIRT2, STAMBP, TNFRSF9	(21, 22, 42, 47, 48, 50, 52, 54, 55, 59, 61, 62)
Long COVID vs active infected	Albumin, CRP, Feritin		G-CSF, IFN-α, IL-1β, IL-6, IL-13, IL-17, IP-10, TNF-α		D-dimer, ESR, Hemoglobin		(55)

A detailed list of abbreviations in Table 2 can be found in Supplementary Table 2.

### Biomarkers in long COVID-19 symptoms

**FIGURE 4:**
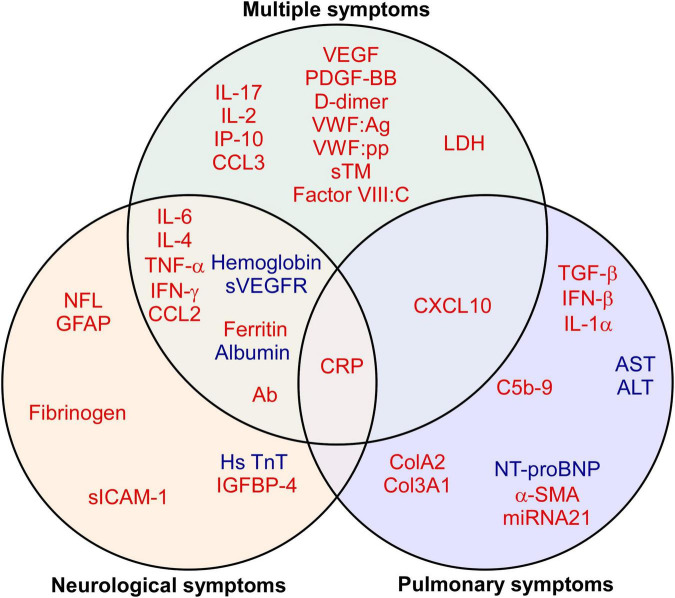
Biomarkers of long COVID symptoms. The Venn diagram presents the 41 biomarkers that were reported by two or more eligible studies. Red indicates up-regulated, while blue refers to down-regulated biomarkers. A detailed list of abbreviations in Figure 4 can be found in Supplementary Table 2.

Among 28 studies, 57.1% (16 of 28) of the studies reported biomarkers in patients with multiple symptoms, followed by 39.3% (11 of 28) with neurological symptoms and 17.9% (5 of 28) with pulmonary symptoms (Table 3). A total of 113 blood biomarkers that were related to long COVID symptoms after SARS-CoV-2 infection, 92 (81.4%), 22 (19.5%), and 15 (13.3%) biomarkers, respectively, showed a significant association with multiple, neurological, and pulmonary symptoms. The major classification of biomarkers was cytokines/chemokines: 35.9% (33 of 92) in multiple symptoms, 22.7% (5 of 22) in neurological symptoms, and 33.3% (5 of 15) in pulmonary symptoms. As shown in Figure 4, through the Venn diagram comparative analysis of these biomarkers, increased CRP was found to be a significant indicator of multiple, neurological, and pulmonary long COVID symptoms. Additionally, several up-regulated vascular biomarkers associated with angiogenesis [VEGF or Platelet derived growth factor BB (PDGF-BB)] and coagulation [D-dimer, von Willebrand factor antigen (VWF:Ag), von Willebrand factor propeptide (VWF:pp), soluble thrombomodulin (sTM), or Factor VIII:C] were reported in patients with multiple symptoms. Elevated neurological biomarkers related to nerve injuries, such as NFL and GFAP, may serve as diagnostic biomarkers for long COVID neurological symptoms, especially for long COVID headaches (19, 20). Moreover, in long COVID pulmonary symptoms, compared with healthy control, long COVID patients with pulmonary fibrosis exhibited higher Transforming growth factor beta (TGF-β) (21, 22). As a result, these biomarkers may serve as indicators of distinct long COVID symptoms.

**TABLE 3:** Biomarkers significantly associated with long COVID Symptoms.

Long COVID symptoms	Categories of biomarkers						References
	Acute phase protein	Biochemical marker	Cytokine/Chemokine	Neurological marker	Vascular marker	Others	
Neurological symptoms	Albumin, CRP, Ferritin, Fibrinogen	β-glucan, S-Sulfocysteine	CCL2, IFN-γ, IL-4, IL-6, TNF-α	GFAP, NFL, pGFAP/pNFL	ET-1, Hemoglobin, sICAM-1, sVCAM-1, sVEGFR	Ab, Hs TnT, IGFBP-4	(19, 20, 43–46, 49, 54, 59, 60, 62)
Pulmonary symptoms	C5b-9, CRP	ALT, AST, LDH	CXCL10, IFN-β, IL-1α, IL-6, TGF-β		Col1A2, Col3A1	α-SMA, miRNA21, NT-proBNP	(21, 22, 40, 46, 51)
Multiple symptoms	Albumin, CRP, Ferritin,	1-Methylnicotinamide, 2-Phenylphenol, 3,5-Dihydroxybenzoic acid, ADA, β-glucan, CPA3, Glutamine/Glutamate ratio, Indole-3-lactic acid, L-Cystein, LDH, L-Glutamine, L-Methionine, Ornithine, Pipecolic acid, Quinolinic acid, Quinolinic acid/Tryptophan ratio, Sarcosine, ST1A1, S-Sulfocysteine, Taurine, Tryptase, uPA	CCL2, CCL3, CCL4, CCL5, CCL7, CCL19, CCL20, CCL23, CXCL1, CXCL9, CXCL10, CXCL11, Flt3L, GM-CSF, IFN-γ, IL-1α, IL-2, IL-4, IL-6, IL-7, IL-10, IL-10Rβ, IL-12β, IL-17, IL-18, IL-33, IP-10, M-CSF, SCF, TGF-α, TGF-β, TNF-α, TNF-β	GDNF, NGF-β, NT-3	Ang-2, D-dimer, ESR, ET-1, Factor VIII:C, Hemoglobin, MMP-1, MMP-9, MPO, PDGF-BB, sVCAM-1, sTM, sVEGFR, VEGF, VWF:Ag, VWF:pp	Ab, ARTN, AXIN, CASP-8, CST-5, Cystatin C, LBP, MRP8/14, NGAL, OPG, OSM, SIRT2, STAMBP, TNFRSF9, Zonulin	(40–42, 47–50, 52, 53, 55–59, 61, 62)
Cardiac symptoms					NO	NT-BNP	(63)

A detailed list of abbreviations in Table 3 can be found in Supplementary Table 2.

## Discussion

Of 193 putative biomarkers tested, 113 were found in this review to be statistically significantly associated with long COVID. To provide a functional view of the biomarkers, we divided the 113 biomarkers into six categories based on their biological function: cytokine/chemokine, biochemical markers, vascular markers, neurological markers, acute phase protein, and others. Through a comprehensive evidence synthesis of biomarkers in long COVID, the up-regulated IL-6, CRP, and TNF-α were found to be a potential core set of biomarkers for long COVID.

### Role of circulating biomarkers in long COVID-associated neurological dysfunction

Severe systemic inflammation and substantial tissue damage in acute COVID are accelerated by pro-inflammatory cytokines/chemokines (23), which involve many pathophysiological mechanisms, like leukocyte trafficking (24), cytokine storm (25), and normal tissue necroptosis (26). Systemic inflammatory markers, such as IL-6 and CRP, were associated with disease severity and mortality among COVID-19 patients (27). Moreover, consistent with our findings (Figures 3, 4), the prolonged IL-6, TNF-α, and CRP were also implicated in systemic and neurological long COVID sequelae (28).

Neurological symptoms are the most common long COVID clinical manifestations (7). NFL and GFAP are skeleton proteins that maintain the stability of neuron axons and astrocytes. The expression of these neural peptides in circulation may serve as biomarkers associated with neuronal degeneration and damage (29, 30). Long COVID patients with elevated serum NFL and GFAP showed worse headaches and persistent neuropathic pain (19, 20). Furthermore, Peluso et al. reported that the serum levels of NFL and GFAP in post-acute COVID patients are positively correlated with IL-6, TNF-α, and CCL2 (19) that may induce immune cells and activate detrimental neuroinflammation (31). This indirect mechanism demonstrates that pro-inflammatory cytokines/chemokines may exacerbate substantial neuronal damage.

### Role of circulating biomarkers in long COVID-associated pulmonary fibrosis

Pulmonary fibrosis is one of the complications of severe COVID cases (32). Similar to the long COVID patients with pulmonary symptoms, elevated levels of IL-6, CRP, and TGF-β were identified in patients at increased risk of developing pulmonary fibrosis after SARS-CoV-2 infection (Figure 4) (33, 34). TGF-β is a multifunctional cytokine that plays a crucial role in tissue repair after injury. Upon a pulmonary viral infection, epithelial cell injury may induce the activation of M2 macrophages to secrete TGF-β, stimulating fibroblast proliferation and collagen synthesis and leading to fibrosis (22, 35). Recently, Zhou et al. demonstrated that Pirfenidone, an Food and Drug Administration (FDA)-approved TGF-β/collagen-targeted drug, attenuated the post-COVID-19 pulmonary fibrosis manifestation (36). Hence, a combination therapy targeting the anti-inflammatory (such as IL-6 blockades) (13) and anti-fibrotic pathways (such as Pirfenidone) (36) may be a potential therapeutical strategy for long COVID with pulmonary fibrosis.

### Future directions toward the use of biomarkers

In this review, we have evaluated and summarized the long COVID-related biomarkers. However, because of the heterogeneity of long COVID, no laboratory test could definitively distinguish long COVID from other diseases. A panel of markers may effectively differentiate long COVID cases from others and serve as potential biomarkers for early detection of long COVID. As shown in Figures 3, 4, in addition to the use of a core set of biomarkers (IL-6, CRP, and TNF-α), IL-4, Interferon (IFN) gamma, CCL2, Ferritin, Hemoglobin, NFL, and GFAP may be added in the penal of long COVID patients with neurological symptoms. Likewise, C-X-C motif chemokine ligand 10 (CXCL10), TGF-β, IFN-β, and IL-1α may be included in the panel in patients with pulmonary symptoms. Holistic patient-centered care and the improved management of long COVID may require the integration of symptom management approaches, the current panel of long COVID biomarkers, as well as additional more specific biomarkers that are yet to be identified. Furthermore, some biomarkers may also be affected by participants’ existing clinical conditions. For example, NFL may serve as not only a biomarker for long COVID neurological symptoms in this study but also a biomarker for neurodegenerative diseases, such as Alzheimer’s disease (10) and Parkinson’s disease (11). Therefore, future application of this panel of long COVID biomarkers may need to consider the patient’s clinical history to avoid concomitant pathologies of other diseases.

### Strengths and weaknesses of the research

Our study highlights the first systematic review to synthesize unique expression patterns of inflammatory biomarkers in long COVID, and assess whether they can serve as diagnostic or prognostic markers. Categorization of the biomarkers into six categories based on biological function may further inform our understanding of the clinicopathology of long COVID. The findings may guide and help clinicians to identify a core set of blood biomarkers that can be used to monitor and manage long COVID in clinical practice. Nevertheless, there are several limitations to our approach. First, as shown in the quality assessment (Supplementary Table 3) of the manuscript, 96.4% (27 of 28) of the eligible articles provided different sampling criteria to exclude participants with some existing disease conditions based on the clinical history of patients. Moreover, most of the biomarkers in the eligible studies were measured after the onset of the long COVID symptoms. Therefore, the main biomarkers found to be overexpressed in long COVID, such as IL-6, CRP, and TNF-α, although important in COVID, likely lack specificity to serve as predictors for long COVID. The identified biomarkers are real and reflect the biology of viral infections which do activate the inflammasome, leading to the production of important cytokines/chemokines (IL-1β, TNF-α, IL-6, etc.) (37, 38). This may be the main reason why existing studies predominantly measured only these main inflammatory biomarkers. Unfortunately, many other stressors, such as viruses, bacteria, inhaled nano/particles, industrial toxins, etc., do share common mechanistic features that involve inflammation via inflammasome activation. For this reason, it is necessary that future studies on long COVID employ broader screening platforms that are likely to yield unique and likely specific biomarkers for SARS-CoV-2. For example, a recent study proposed that IL-26 may be a COVID-19-specific biomarker, but none of the studies to date have measured IL-26 (39). Furthermore, there is a lack of consistency on specific long COVID symptoms. 57.1% of the eligible studies examined biomarkers for patients with multiple symptoms of long COVID. Additionally, the duration of facing persistent long COVID symptoms varied within and across studies. Incongruent with long COVID symptoms and timeframes may contribute to distinct biomarkers. Finally, vaccination may affect patients’ physiological variables and the levels of biomarkers in serum. However, there is no sufficient evidence to know the effect of vaccination because only one of the 28 eligible studies separated the vaccinated participants from the unvaccinated ones. Such issues should be addressed in future longitudinal studies on biomarker expression among long COVID patients to understand the causal relationship between long COVID symptom development and acute COVID inflammation.

## Summary

Long COVID patients present elevated inflammatory biomarkers after initial infection. Our study found that people with higher levels of IL-6, CRP, and TNF-α after SARS-CoV-2 infection for one or more months may experience long-term COVID symptoms. This systematic review could identify a panel of blood biomarkers that can be used to manage long COVID patients in clinical practice.

## Data availability statement

The original contributions presented in this study are included in the article/Supplementary material, further inquiries can be directed to the corresponding author.

## Author contributions

Y-JL supervised the entire project, designed, analyzed manuscripts and wrote manuscript. S-HL and SM designed, analyzed manuscripts, and wrote manuscript. T-AL analyzed manuscripts and wrote manuscript. C-TK analyzed manuscripts. DB provided scientific input and wrote the manuscript. All authors contributed to the article and approved the submitted version.
