# Supplementary material for: Biomarkers in long COVID-19: A systematic review
Source: Front Med (Lausanne). 2023 Jan 20;10:1085988. doi: 10.3389/fmed.2023.1085988 (PMC9895110; doi:10.3389/fmed.2023.1085988)
Supplement: Supplementary file 1 [file Table_1.pdf]

Supplementary Table 1. Search strategy and search results

Database: PubMed

Search date: 08/18/2022

	Group	Keyword	Results
1	long COVID-19 symptoms	a. COVID-19<br>"SARS-CoV-2"[MeSH Terms] OR "COVID-19"[MeSH Terms] OR "Severe Acute Respiratory Syndrome Coronavirus 2"[Text Word] OR "SARS-CoV-2"[Text Word] OR "SARS-CoV2"[Text Word] OR "COVID-19"[Text Word] OR "COVID 19"[Text Word] OR "2019 novel coronavirus"[Text Word] OR "2019 Novel Coronaviruses"[Text Word] OR "2019 nCoV"[Text Word] OR "n-CoV"[Text Word] OR "Wuhan coronavirus"[Text Word] OR "Coronavirus Disease 2019"[Text Word] OR "Coronavirus Disease-19"[Text Word] OR "Coronavirus Disease 19"[Text Word] OR "SARS Coronavirus 2"[Text Word]	281,414
		b. Symptoms<br>"symptom*"[Text Word] OR "clinical features"[Text Word] OR "signs"[Text Word] OR "characteristic*"[Text Word] OR "sequela*"[Text Word] OR "complication*"[Text Word] OR "manifestation*"[Text Word] OR "indication*"[Text Word] OR "syndrome*"[Text Word] OR "clinical significance"[Text Word] OR "clinical significant"[Text Word] OR "hallmarks*"[Text Word] OR "clinical presentations"[Text Word]	7,349,823
		c. Long COVID<br>"post-acute COVID-19 syndrome" [Supplementary Concept] OR "post-acute COVID-19 syndrome"[Text Word] OR "long COVID-19 symptoms"[Text Word] OR "long COVID"[Text Word] OR "long-COVID"[Text Word] OR "long-haul COVID"[Text Word] OR "long haul COVID"[Text Word] OR "post COVID-19 syndromes"[Text Word] OR "post COVID-19 syndrome"[Text Word] OR "post-acute COVID syndrome"[Text Word] OR "post-acute COVID19 syndrome"[Text Word] OR "persistent COVID-19"[Text Word] OR "long hauler"[Text Word] OR "post-acute sequelae of SARS-Cov-2 infection"[Text Word] OR "post COVID-19 conditions"[Text Word] OR "post-COVID-19 condition"[Text Word] OR "post-acute sequelae of COVID-19"[Text Word] OR "chronic COVID syndrome"[Text Word]	11,272

	Group	Keyword	Results
		OR "PASC"[Text Word] OR "CCS"[Text Word] OR "COVID-19 recovery"[Text Word] OR "COVID 19 recovery"[Text Word] OR "Coronavirus recovery"[Text Word] OR "COVID 19 recovery"[Text Word] OR "post-recovery symptoms"[Text Word] OR "recovered COVID 19"[Text Word] OR "post-discharge COVID 19"[Text Word] OR "post-COVID-19 manifestations"[Text Word] OR "post-COVID-19 manifestation"[Text Word] OR "post COVID manifestations"[Text Word] OR "post COVID manifestation"[Text Word] OR "post-COVID-19 rehabilitation"[Text Word] OR "COVID 19 survival"[Text Word] OR "post-COVID-19 complications"[Text Word] OR "post-COVID-19 complication"[Text Word]	
		**a AND b AND c**	2,558
2	Biomarkers	"biomarkers"[MeSH Terms] OR "biomarkers"[Text Word] OR "biomarker"[Text Word] OR "cytokines"[MeSH Terms] OR "cytokines"[Text Word] OR "cytokine"[Text Word] OR "chemokines"[MeSH Terms] OR "chemokines"[Text Word] OR "chemokine"[Text Word] OR "immune markers"[Text Word] OR "immune marker"[Text Word] OR "immunologic markers"[Text Word] OR "immunologic marker"[Text Word] OR "blood biomarker"[Text Word] OR "blood biomarkers"[Text Word] OR "serum biomarker"[Text Word] OR "serum biomarkers"[Text Word] OR "plasma biomarker"[Text Word] OR "plasma biomarkers"[Text Word] OR "plasma marker"[Text Word] OR "plasma markers"[Text Word] OR "serum marker"[Text Word] OR "serum markers"[Text Word] OR "blood marker"[Text Word] OR "blood markers"[Text Word] OR "blood indicator"[Text Word] OR "blood indicators"[Text Word] OR "serum indicator"[Text Word] OR "serum indicators"[Text Word] OR "plasma indicator"[Text Word] OR "plasma indicators"[Text Word] OR "plasma factors"[Text Word] OR "plasma factor"[Text Word] OR "serum factors"[Text Word] OR "serum factor"[Text Word] OR "blood factors"[Text Word] OR "blood factor"[Text Word] OR "biological marker"[Text Word] OR "biological markers"[Text Word] OR "biologic marker"[Text Word] OR "biologic markers"[Text Word] OR "laboratory markers"[Text Word] OR "laboratory marker"[Text Word]	1,899,335
	**1+2**	**1 AND 2**	**265**

Database: CINAHL

Search date: 08/18/2022

	Group	Keyword	Results
1	long COVID-19 symptoms	a. COVID-19<br>(MH "SARS-CoV-2") OR (MH "COVID-19") OR "Severe Acute Respiratory Syndrome Coronavirus 2" OR "SARS-CoV-2" OR "SARS-CoV2" OR "COVID-19" OR "COVID 19" OR "2019 novel coronavirus" OR "2019 Novel Coronaviruses" OR "2019 nCoV" OR "n-CoV" OR "Wuhan coronavirus" OR "Coronavirus Disease 2019" OR "Coronavirus Disease-19" OR "Coronavirus Disease 19" OR "SARS Coronavirus 2"	106,376
		b. Symptoms<br>"symptom*" OR "clinical features" OR "signs" OR "characteristic*" OR "sequela*" OR "complication*" OR "manifestation*" OR "indication*" OR "syndrome*" OR "clinical significance" OR "clinical significant" OR "hallmarks*" OR "clinical presentations"	1,640,718
		c. Long COVID<br>"post-acute COVID-19 syndrome" OR "long COVID-19 symptoms" OR "long COVID" OR "long-COVID" OR "long-haul COVID" OR "long haul COVID" OR "post COVID-19 syndromes" OR "post COVID-19 syndrome" OR "post-acute COVID syndrome" OR "post-acute COVID19 syndrome" OR "persistent COVID-19" OR "long hauler" OR "post-acute sequelae of SARS-Cov-2 infection" OR "post COVID-19 conditions" OR "post-COVID-19 condition" OR "post-acute sequelae of COVID-19" OR "chronic COVID syndrome" OR "PASC" OR "CCS" OR "COVID-19 recovery" OR "COVID 19 recovery" OR "Coronavirus recovery" OR "COVID 19 recovery" OR "post-recovery symptoms" OR "recovered COVID 19" OR "post-discharge COVID 19" OR "post-COVID-19 manifestations" OR "post-COVID-19 manifestation" OR "post COVID manifestations" OR "post COVID manifestation" OR "post-COVID-19 rehabilitation" OR "COVID 19 survival" OR "post-COVID-19 complications" OR "post-COVID-19 complication"	2,397
		**a AND b AND c**	622
2	Biomarkers	(MH "biomarkers") OR "biomarkers" OR "biomarker" OR (MH "cytokines") OR "cytokines" OR "cytokine" OR (MH	157,389

Group	Keyword	Results
	"chemokines") OR "chemokines" OR "chemokine" OR "immune markers" OR "immune marker" OR "immunologic markers" OR "immunologic marker" OR "blood biomarker" OR "blood biomarkers" OR "serum biomarker" OR "serum biomarkers" OR "plasma biomarker" OR "plasma biomarkers" OR "plasma marker" OR "plasma markers" OR "serum marker" OR "serum markers" OR "blood marker" OR "blood markers" OR "blood indicator" OR "blood indicators" OR "serum indicator" OR "serum indicators" OR "plasma indicator" OR "plasma indicators" OR "plasma factors" OR "plasma factor" OR "serum factors" OR "serum factor" OR "blood factors" OR "blood factor" OR "biological marker" OR "biological markers" OR "biologic marker" OR "biologic markers" OR "laboratory markers" OR "laboratory marker"	
**1+2**	**1 AND 2**	**23**

Database: Embase

Search date: 08/18/2022

	Group	Keyword	Results
1	long COVID-19 symptoms	a. COVID-19<br>‘SARS-CoV-2’/exp/mj OR ‘COVID-19’/exp/mj OR ‘Severe Acute Respiratory Syndrome Coronavirus 2’:ti,ab,kw OR ‘SARS-CoV-2’:ti,ab,kw OR ‘SARS-CoV2’:ti,ab,kw OR ‘COVID-19’:ti,ab,kw OR ‘COVID 19’:ti,ab,kw OR ‘2019 novel coronavirus’:ti,ab,kw OR ‘2019 Novel Coronaviruses’:ti,ab,kw OR ‘2019 nCoV’:ti,ab,kw OR ‘n-CoV’:ti,ab,kw OR ‘Wuhan coronavirus’:ti,ab,kw OR ‘Coronavirus Disease 2019’:ti,ab,kw OR ‘Coronavirus Disease-19’:ti,ab,kw OR ‘Coronavirus Disease 19’:ti,ab,kw OR ‘SARS Coronavirus 2’:ti,ab,kw	301,800
		b. Symptoms<br>‘symptom*’:ti,ab,kw OR ‘clinical features’:ti,ab,kw OR ‘signs’:ti,ab,kw OR ‘characteristic*’:ti,ab,kw OR ‘sequela*’:ti,ab,kw OR ‘complication*’:ti,ab,kw OR ‘manifestation*’:ti,ab,kw OR ‘indication*’:ti,ab,kw OR ‘syndrome*’:ti,ab,kw OR ‘clinical significance’:ti,ab,kw OR ‘clinical significant’:ti,ab,kw OR ‘hallmarks*’:ti,ab,kw OR ‘clinical presentations’:ti,ab,kw	7,359,993
		a. Long COVID<br>‘post-acute COVID-19 syndrome‘:ti,ab,kw OR ‘long COVID-19 symptoms’:ti,ab,kw OR 'long covid'/exp OR ‘long COVID’:ti,ab,kw OR ‘long-COVID’:ti,ab,kw OR ‘long-haul COVID’:ti,ab,kw OR ‘long haul COVID’:ti,ab,kw OR ‘post COVID-19 syndromes’:ti,ab,kw OR ‘post COVID-19 syndrome’:ti,ab,kw OR ‘post-acute COVID syndrome’:ti,ab,kw OR ‘post-acute COVID19 syndrome’:ti,ab,kw OR ‘persistent COVID-19’:ti,ab,kw OR ‘long hauler’:ti,ab,kw OR ‘post-acute sequelae of SARS-Cov-2 infection’:ti,ab,kw OR ‘post COVID-19 conditions’:ti,ab,kw OR ‘post-COVID-19 condition’:ti,ab,kw OR ‘post-acute sequelae of COVID-19’:ti,ab,kw OR ‘chronic COVID syndrome’:ti,ab,kw OR ‘PASC’:ti,ab,kw OR ‘CCS’:ti,ab,kw OR ‘COVID-19 recovery’:ti,ab,kw OR ‘COVID 19 recovery’:ti,ab,kw OR ‘Coronavirus recovery’:ti,ab,kw OR ‘COVID 19 recovery’:ti,ab,kw OR ‘post-recovery symptoms’:ti,ab,kw OR ‘recovered COVID 19’:ti,ab,kw OR ‘post-discharge COVID 19’:ti,ab,kw OR ‘post-COVID-19 manifestations’:ti,ab,kw OR ‘post-COVID-	16,914

	Group	Keyword	Results
		19 manifestation':ti,ab,kw OR 'post COVID manifestations':ti,ab,kw OR 'post COVID manifestation':ti,ab,kw OR 'post-COVID-19 rehabilitation':ti,ab,kw OR 'COVID 19 survival':ti,ab,kw OR 'post-COVID-19 complications':ti,ab,kw OR 'post-COVID-19 complication':ti,ab,kw<br><br>**a AND b AND c**	2,946
2	Biomarkers	'biomarkers'/exp/mj OR 'biomarkers':ti,ab,kw OR 'biomarker':ti,ab,kw OR 'cytokines'/exp/mj OR 'cytokines':ti,ab,kw OR 'cytokine':ti,ab,kw OR 'chemokines'/exp/mj OR 'chemokines':ti,ab,kw OR 'chemokine':ti,ab,kw OR 'immune markers':ti,ab,kw OR 'immune marker':ti,ab,kw OR 'immunologic markers':ti,ab,kw OR 'immunologic marker':ti,ab,kw OR 'blood biomarker':ti,ab,kw OR 'blood biomarkers':ti,ab,kw OR 'serum biomarker':ti,ab,kw OR 'serum biomarkers':ti,ab,kw OR 'plasma biomarker':ti,ab,kw OR 'plasma biomarkers':ti,ab,kw OR 'plasma marker':ti,ab,kw OR 'plasma markers':ti,ab,kw OR 'serum marker':ti,ab,kw OR 'serum markers':ti,ab,kw OR 'blood marker':ti,ab,kw OR 'blood markers':ti,ab,kw OR 'blood indicator':ti,ab,kw OR 'blood indicators':ti,ab,kw OR 'serum indicator':ti,ab,kw OR 'serum indicators':ti,ab,kw OR 'plasma indicator':ti,ab,kw OR 'plasma indicators':ti,ab,kw OR 'plasma factors':ti,ab,kw OR 'plasma factor':ti,ab,kw OR 'serum factors':ti,ab,kw OR 'serum factor':ti,ab,kw OR 'blood factors':ti,ab,kw OR 'blood factor':ti,ab,kw OR 'biological marker':ti,ab,kw OR 'biological markers':ti,ab,kw OR 'biologic marker':ti,ab,kw OR 'biologic markers':ti,ab,kw OR 'laboratory markers':ti,ab,kw OR 'laboratory marker':ti,ab,kw	1,645,283
	**1+2**	**1 AND 2**	**286**
