# Supplementary material for: Biomarkers in long COVID-19: A systematic review
Source: Front Med (Lausanne). 2023 Jan 20;10:1085988. doi: 10.3389/fmed.2023.1085988 (PMC9895110; doi:10.3389/fmed.2023.1085988)
Supplement: Supplementary file 3 [file Table_3.pdf]

Supplemental Table 3. Quality Assessment of Each Study Included in the Systematic Review by Using the Modified REMARK Questionnaire

Article	Item of REMARK questionnaire								Percentage of Yes
	1	2	3	4	5	6	7	8	
1	Yes	No	No	No	Yes	No	Yes	Yes	50
2	Yes	No	No	No	Yes	No	Yes	Yes	50
3	Yes	No	Yes	Yes	Yes	Yes	Yes	Yes	87.5
4	No	No	Yes	Yes	Yes	Yes	Yes	Yes	75
5	Yes	No	No	Yes	Yes	No	Yes	Yes	62.5
6	Yes	No	Yes	No	Yes	Yes	No	Yes	62.5
7	Yes	No	No	Yes	Yes	Yes	No	Yes	62.5
8	Yes	No	Yes	Yes	Yes	Yes	Yes	Yes	87.5
9	Yes	No	Yes	No	Yes	No	Yes	Yes	62.5
10	Yes	No	No	Yes	Yes	No	Yes	Yes	62.5
11	Yes	No	No	Yes	Yes	Yes	Yes	Yes	75
12	Yes	No	Yes	Yes	Yes	Yes	Yes	Yes	87.5
13	Yes	No	No	No	Yes	No	Yes	Yes	50
14	Yes	No	Yes	Yes	Yes	No	Yes	Yes	75
15	Yes	No	Yes	Yes	Yes	Yes	Yes	Yes	87.5
16	Yes	No	Yes	Yes	Yes	Yes	Yes	Yes	87.5
17	Yes	No	Yes	Yes	Yes	Yes	No	Yes	75
18	Yes	No	Yes	No	Yes	Yes	Yes	Yes	75
19	Yes	Yes	Yes	Yes	No	Yes	No	Yes	75
20	Yes	No	No	Yes	Yes	No	Yes	No	50
21	Yes	Yes	No	Yes	Yes	No	Yes	Yes	75
22	Yes	Yes	No	Yes	Yes	No	Yes	Yes	75
23	Yes	No	Yes	Yes	Yes	Yes	Yes	Yes	87.5
24	Yes	No	Yes	Yes	Yes	Yes	Yes	Yes	87.5
25	Yes	No	Yes	No	Yes	Yes	Yes	Yes	75
26	Yes	No	Yes	Yes	Yes	Yes	Yes	Yes	87.5
27	Yes	No	No	No	Yes	No	No	Yes	37.5
28	Yes	No	No	No	Yes	Yes	Yes	Yes	62.5
Percentage of Yes	96.4	10.7	57.1	67.9	96.4	60.7	82.1	96.4	
